# A voting-based machine learning approach for classifying biological and clinical datasets

**DOI:** 10.1186/s12859-023-05274-4

**Published:** 2023-04-11

**Authors:** Negar Hossein-Nezhad Daneshvar, Yosef Masoudi-Sobhanzadeh, Yadollah Omidi

**Affiliations:** 1 Department of Computer Engineering, University College of Nabi Akram, Tabriz, Iran; 2 Research Center for Pharmaceutical Nanotechnology, Biomedicine Institute, Tabriz University of Medical Sciences, Tabriz, Iran; 3 Faculty of Advanced Medical Sciences, Tabriz University of Medical Sciences, Tabriz, Iran; 4 Department of Pharmaceutical Sciences, College of Pharmacy, Nova Southeastern University, Florida, 33328 USA

**Keywords:** Clinical datasets, Feature selection, Gene selection, Machine learning, Optimization algorithm, Voting-based approach

## Abstract

**Background:**

Different machine learning techniques have been proposed to classify a wide range of biological/clinical data. Given the practicability of these approaches accordingly, various software packages have been also designed and developed. However, the existing methods suffer from several limitations such as overfitting on a specific dataset, ignoring the feature selection concept in the preprocessing step, and losing their performance on large-size datasets. To tackle the mentioned restrictions, in this study, we introduced a machine learning framework consisting of two main steps. First, our previously suggested optimization algorithm (*Trader*) was extended to select a near-optimal subset of features/genes. Second, a voting-based framework was proposed to classify the biological/clinical data with high accuracy. To evaluate the efficiency of the proposed method, it was applied to 13 biological/clinical datasets, and the outcomes were comprehensively compared with the prior methods.

**Results:**

The results demonstrated that the *Trader* algorithm could select a near-optimal subset of features with a significant level of p-value < 0.01 relative to the compared algorithms. Additionally, on the large-sie datasets, the proposed machine learning framework improved prior studies by ~ 10% in terms of the mean values associated with fivefold cross-validation of accuracy, precision, recall, specificity, and F-measure.

**Conclusion:**

Based on the obtained results, it can be concluded that a proper configuration of efficient algorithms and methods can increase the prediction power of machine learning approaches and help researchers in designing practical diagnosis health care systems and offering effective treatment plans.

## Background

Classification is the process of dividing data samples into different groups using the machine learning (ML) approaches [1]. This technique has been extended to a wide range of computational and biological applications such as identifying potential gene/miRNA/protein biomarkers [2], repurposing drugs against different diseases [3], suggesting novel therapeutic modalities for curing illnesses [4], diagnosing heart and diabetes sicknesses [5], and better perceiving biological phenomena [6]. In this line, several ML strategies have been developed, resulting in the generation of computer-aided health decision support systems [7]. These strategies aimed to improve the ML and feature selection (FS) algorithms mainly because of their effects on the performance of a classification model [8]. For instance, to diagnose diabetes disease in its early stages, Patil et al*.* utilized C4.5 and k-means clustering ML algorithms and achieved ~ 92.38% value of tenfold cross-validation accuracy on the Pima Indian Diabetes (PID) dataset [9]. To this end, the researchers removed serum insulin and triceps skinfold features and reduced the total number of samples from 768 to 625. The researchers then determined the data patterns using the k-means algorithm and eliminated 192 other instances. Based on the obtained patterns, a decision tree was formed, and the produced model was evaluated. Although these researchers generated a proper prediction model with a high value of accuracy on the PID dataset, their methods suffered from overfitting because of removing a remarkable number of the data instances. To tackle such a limitation, Aslam et al*.* examined a three-step ML method [10]. In the first phase, based on different statistical methods (e.g., Kolmogorov–Smirnov test and t-test), the existing diabetes features were ranked, and some subsets of diabetes features were produced using a progressive FS manner. In the second phase, for every generated subset of features, a genetic programming technique was employed. In the third phase, the usefulness of the produced features was measured using the k-nearest neighbor (KNN) and support vector machine (SVM) classifiers. The results demonstrated that the Gaussian process-SVM (GP-SVM) technique resulted in about 87% of accuracy. In addition to the PID dataset, several studies targeted other biological/clinical datasets and suggested some real-world consistent prediction models [11–13]. For this purpose, the prior studies combined various computational techniques such as the teaching learning-based optimization algorithm (OA) with the fuzzy wavelet neural network [14], the rough set theory with the backpropagation neural network [15], and the fuzzy concept with the min–max neural networks [16]. The mentioned computational strategies have been applied to the Cleveland heart disease (CHD) [17], Statlog heart disease (SHD) [18], Wisconsin diagnostic breast cancer (WDBC) disease and mammogram datasets [19, 20], respectively. Some researchers also designed other types of hybrid ML techniques and applied them to different biological/clinical datasets [21–24]. The mentioned studies encounter several limitations, including low prediction power, inability in grouping multiclass data (more than two classes), overfitting, and filtering the samples with missing values. Therefore, Arabi et al*.* suggested a ML approach that creates a specific model for every class of existing data [25, 26]. For this purpose, the researchers acquired several regression and classification datasets from the ML repository of the University of California, Irvine (UCI). Then, after normalizing the obtained data, a distinct model was generated for every class of data. For example, if the data of interest included three classes, three individual machines were designed for each of them. In the next phase, a cascade-like artificial neural network was designed and trained using the world competitive contests (WCC) optimization algorithm [27]. The performance of the methods was investigated on the different partitioned train and test datasets (e.g., 70%-30% or 60%-40%), indicating that their methods outperformed other ML approaches in terms of classification criteria. Although the described technique by Arabi and coworkers yielded a model with a higher prediction ability, their ML method was not suitable for large-size datasets.

To address the above-mentioned constraints, in the present study, we extended the *Trader* optimization algorithm for selecting a near-optimal subset of features and generating an efficient prediction model in terms of classification criteria [28]. Additionally, to get better prediction results, a voting-based ML framework was proposed, labeling data samples based on the consensus of predictions obtained from different artificial machines. To evaluate the proposed machine learning approaches, in all the computational experiments, the fivefold cross-validation technique was used.

## Methods

**Fig. 1:**
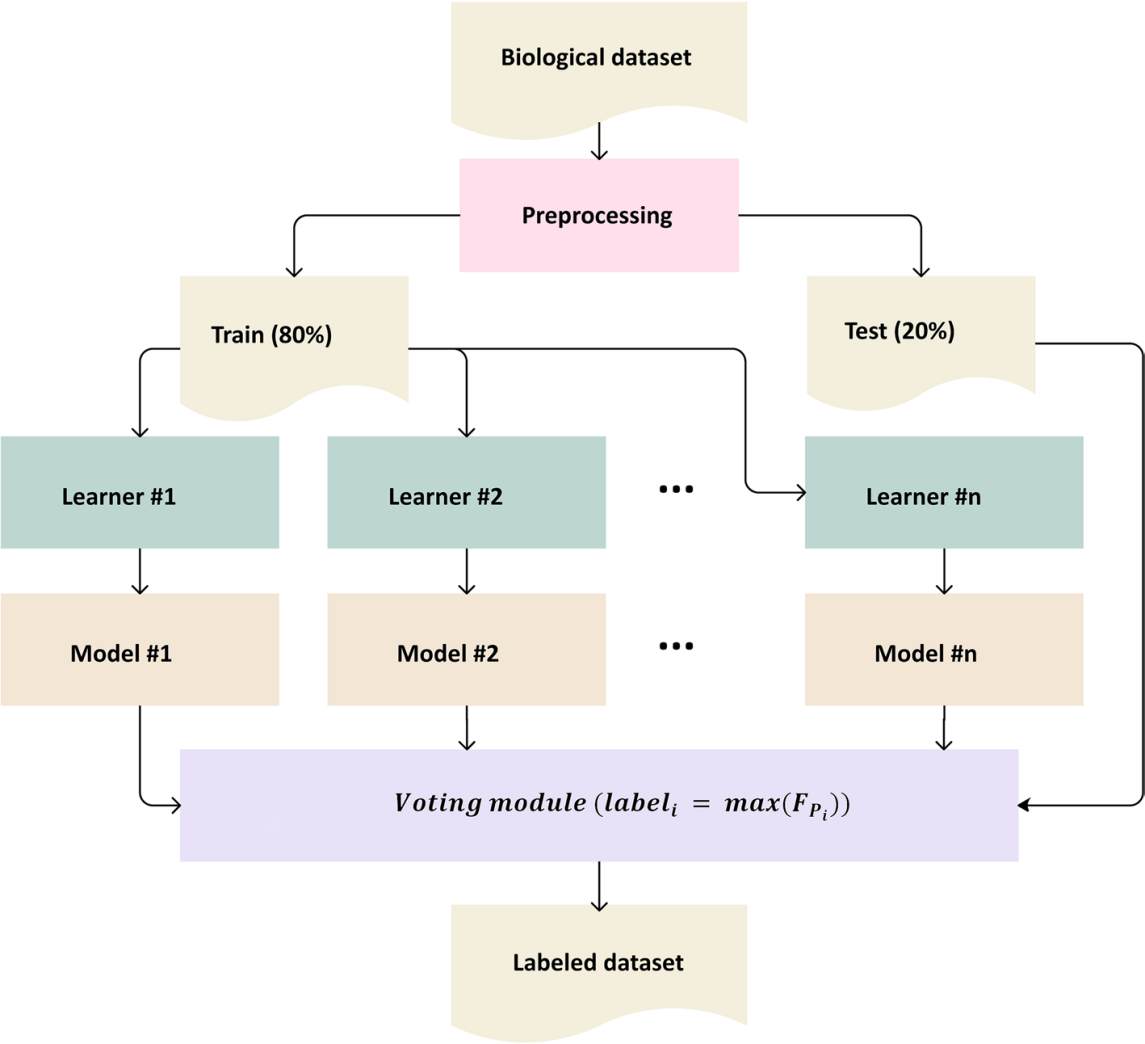
The framework of the proposed voting-based machine learning method for classifying biological/clinical datasets. The final prediction is determined by aggregating the outputs of different models. ${\mathrm{F}}_{{\mathrm{p}}_{\mathrm{i}}}$ shows the total number of models that predicted a given sample belongs to the i^th^ group

The UCI repository has collected various datasets from different scopes and provided a suitable resource for machine learning applications. From this repository, a total of 13 clinical/biological datasets, utilized in various research work as gold-standard input files, were obtained (Table 1). These datasets included different numbers of samples and features/genes, so they seemed to be qualified for evaluating the proposed method in different conditions. The proposed framework, shown in Fig. 1, was applied to these data, and the results were compared from a wide range of classification measurements.

**Table 1:** The properties of the datasets obtained from the UCI repository

Dataset name	#instances	#features	#classes	Data type	Missing values
LIV	345	6	2	Numerical and binary	NO
PID	768	8	2	numerical	NO
SHD	270	13	2	Numerical and binary	NO
CHD2	303	13	2	Numerical and binary	NO
CHD5	303	13	5	Numerical and binary	NO
HEP	150	19	2	Numerical and binary	YES
PAR	197	22	2	Real	YES
WDBC	569	31	2	Real	NO
LUNG	32	56	3	Numerical and binary	YES
ARRYTM	452	279	16	Double	YES
PARKINSON	756	754	2	Numerical and binary	NO
ARCENE	900	10,000	2	Numerical	NO
GENEEXPR	801	20,531	5	Double	NO

The acquired data were preprocessed in two phases that completed missing values and normalized the data, sequentially. To complete the missing values of a given sample, firstly, ten other samples (not including the missing values and sharing a high value of Pearson correlation coefficient (> 0.5) with the sample of interest) were chosen if plausible. Secondly, the missing value of the sample of interest was determined based on calculating the interpolation of the related values. Finally, for normalizing the data, Eq. 1 was used, which converts the data of a feature to range [0, 1].

1 $$x_{{i.j_{new} }} = \frac{{x_{{i.j_{current} - min_{j} }} }}{{max_{j} - min_{j} }}$$

where *x*_*i,j*_*, min*_*j*_*,* and *max*_*j*_ represent the value of the i^th^ row and j^th^ column of a given data, and the minimum and maximum values of the j^th^ column, respectively.

**Fig. 2:**
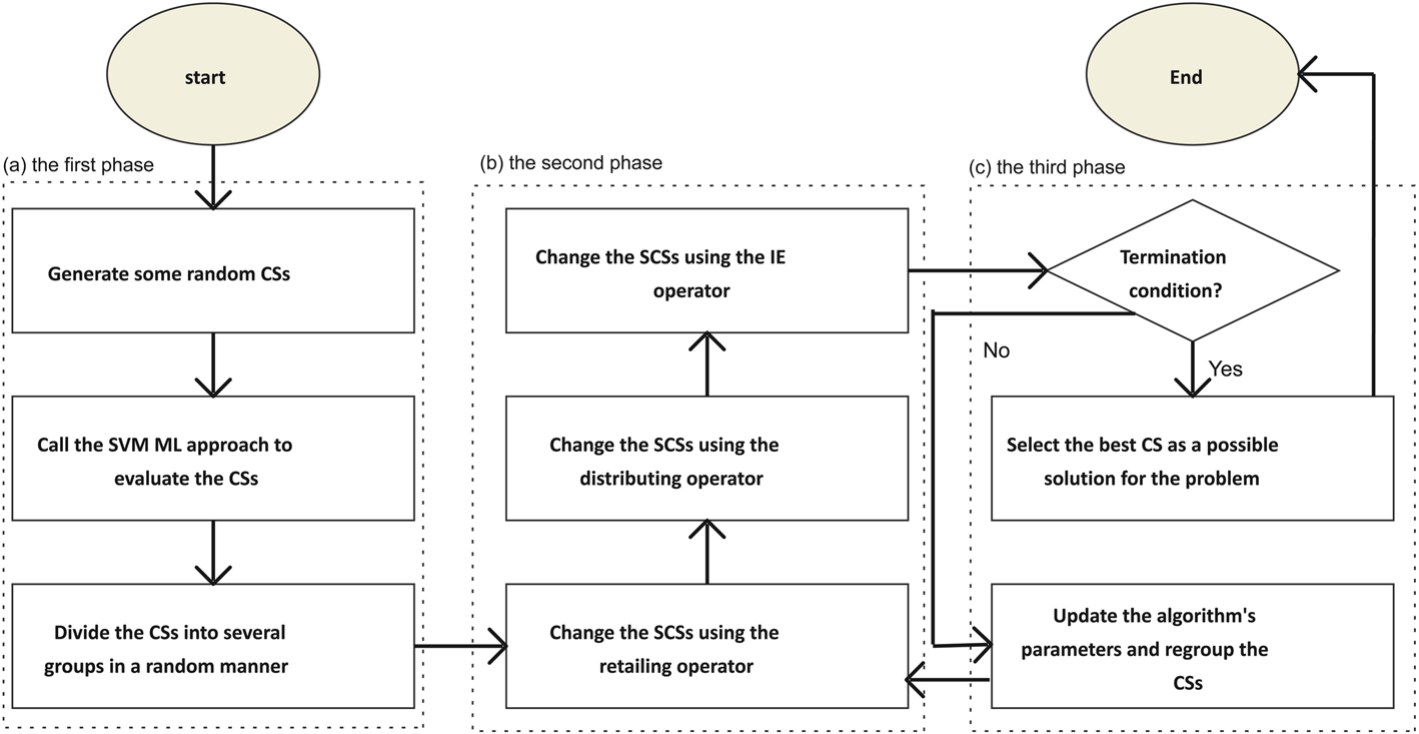
The flowchart of the suggested *Trader* optimization algorithm for selecting a near-optimal subset of features/genes. The algorithm generates some random candidate solutions (CS) and evaluates them using the value of accuracy obtained from the support vector machine (SVM) classifier. Next, the algorithm divides the CSs into several groups and modifies them with three operators. At the final step, the best acquired CS is introduced as a near-optimal subset of features/genes, which can enhance the prediction ability of the SVM classifier

Our previously suggested *Trader* optimization algorithm (OA) was modified and developed to select an optimal/near-optimal subset of features/genes [29, 30]. In this line, as shown in Fig. 2, the algorithm generated some potential candidate solutions (CS) randomly, each of which included a set of selected features/genes and was displayed using an array (Eq. 2).

2 $${\text{CS}} = \left[ {{\text{V}}_{{1}} ,{\text{V}}_{{2}} ,{\text{V}}_{{3}} , \ldots ,{\text{V}}_{{\text{n}}} } \right]$$

where *CS* and *V*_*i*_ stand for a candidate solution and its i^th^ variable, respectively.

The CSs were then divided into several groups in a random manner. Every group and the sum of its CSs’ scores represented a trader and its finances/benefits, respectively.

In the next step, the produced CSs were evaluated using the SVM classification technique (i.e., the accuracy obtained from SVM, was considered as the worthiness/fitness/score of a given subset of features). Since some of the datasets consisted of more than two classes, to calculate the value of accuracy, a mean-based method (Eq. 3) was used.

3 $$Accuracy = \frac{{\mathop \sum \nolimits_{i = 1}^{C} TP_{i} }}{N}$$

where *TP*_*i*_*, C,* and *N* show the total number of true positives of the i^th^ class, the total number of data classes, and the total number of data samples, respectively.

Based on the calculated fitness values, the CSs of every group were separated into master and slave CSs (MCS and SCS, respectively), consequentially showing the best CS and the remaining CSs of a group. Next, the CSs went through a series of changes using the three operators of the *Trader* algorithm, and new CSs were produced. The first operator of *Trader* (the retailing operator (Eq. 4)) created minor modifications in the SCS. This operator usually plays an essential role in improving the CSs in the last steps of the algorithm.

4 $$\forall_{j \in S} \left( {SCS_{i,j} = SCS_{i,j} + k \times random\left( {0 , SCS_{i,j} } \right)} \right)$$

where *SCS*_*i,j*_ and *k* indicate the j^th^ variable of the i^th^ slave CS and a random value (i.e., either -1 or 1), respectively. Additionally, *S* displays a set of randomly selected variables of the i^th^ slave CS.

The second operator of *Trader* (the distributing operator (Eq. 5)) aimed to improve the SCSs by transferring data from their related MCSs. For this purpose, for a given SCS, some variables were randomly chosen from an MCS, and then, their values were transferred to that SCS.

5 $$\forall_{j \in S} \left( {SCS_{i,j} = MCS_{i,j} } \right)$$

where *SCS*_*i,j*_*, MCS*_*i,j*_*,* and *S* are the j^th^ variable of the i^th^ SCS, the j^th^ variable of the i^th^ group’s MCS*,* and a set of randomly chosen variables of the i^th^ CS, respectively.

The third operator of *Trader*, the importing-exporting (IE) operator (Eq. 6), aimed to correct the MCSs and change CSs globally. For this purpose, an MCS was considered the importer while the others as exporters. Like two previous operators, the IE operator acted randomly and changed a given MCS in a similar method described for the second operator.

6 $$\forall_{j \in S, k \in M} \left( {MCS_{i,j} = MCS_{k,j} } \right)$$

where *MCS*_*i,j*_*, MCS*_*M,j*_*, S,* and *M* display the j^th^ variable of the i^th^ importer MCS, the j^th^ variable of the M^th^ exporter MCS, a set of randomly chosen variables of the i^th^ importer MCS, and a set of randomly chosen variables of the M^th^ exporter MCS, respectively.

The proposed OA was compared with other popular OAs in the same conditions. For instance, all the algorithms had a time order of O(n^3^) and called an identical number of the objective function (SVM) during a distinct run.

## Results

The proposed method was implemented in the MATLAB programming language, and the outputs were examined in terms of various criteria associated with evaluating the modified optimization algorithm (*Trader*) and proposed voting-based classification system. To evaluate the usefulness of *Trader* in selecting informative features/genes, it was applied to the downloaded datasets (Table 1), and the outcomes were compared with four other public/effective optimization algorithms. These algorithms (i.e., WCC [27], LCA [31], PSO [32], and ICA [33]) were chosen because of their diversities and proper functionalities reported in the prior studies. Because the values of OAs’ parameters strongly affected their efficiencies, a trial–error method was employed to regulate them [34]. The initial population size of the algorithms was set to 100, and, their steps iteration parameter was regulated to 50. Besides, in every iteration, each of the algorithms changed 30% of candidate solutions. The results of this section were organized into three sections described as follows.

**Fig. 3:**
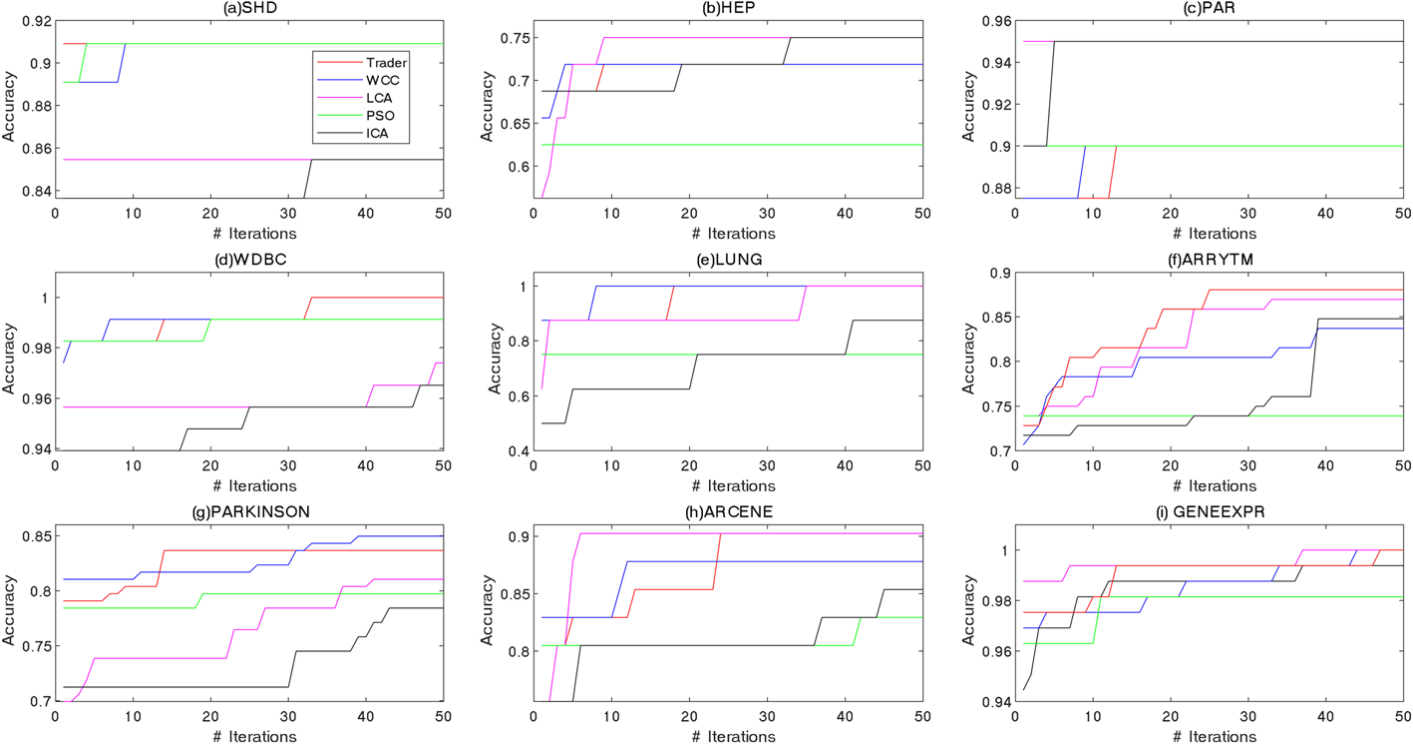
The convergence behavior of the OAs on the **a** SHD, **b** HEP, **c** PAR, **d** WDBC, **e** LUNG, **f** ARRYTM, **g** PARKINSON, **h** ARCENE, and **i** GENEEXPR datasets

The first part of the results compared the performance of the mentioned OAs in terms of improving the prediction power of a learner. To this end, the data of features/genes, chosen by the algorithms, were passed to SVM [35], and the learner then created a model for classifying them. As mentioned in the materials and methods section, OAs generated some random potential answers and modified/improved them using their operators. Hence, it was usually expected to get better results in the j^th^ iteration than in the i^th^ iteration (j > i) (the convergence behavior of OAs) [36]. For the datasets consisting of > 10 features/genes, the convergence behavior of the algorithm was followed, and the mean outcomes of 50 individual executions, were depicted (Fig. 3). Since the performance of the algorithms on the SHD and CHD datasets were similar, only the convergence diagram of the algorithms on the SHD was displayed. Based on the acquired results, *Trader* was able to select more distinctive features and get higher values of accuracy than the other algorithms. Therefore, it can be articulated that the proposed OA had a better convergence behavior than other compared OAs.

**Fig. 4:**
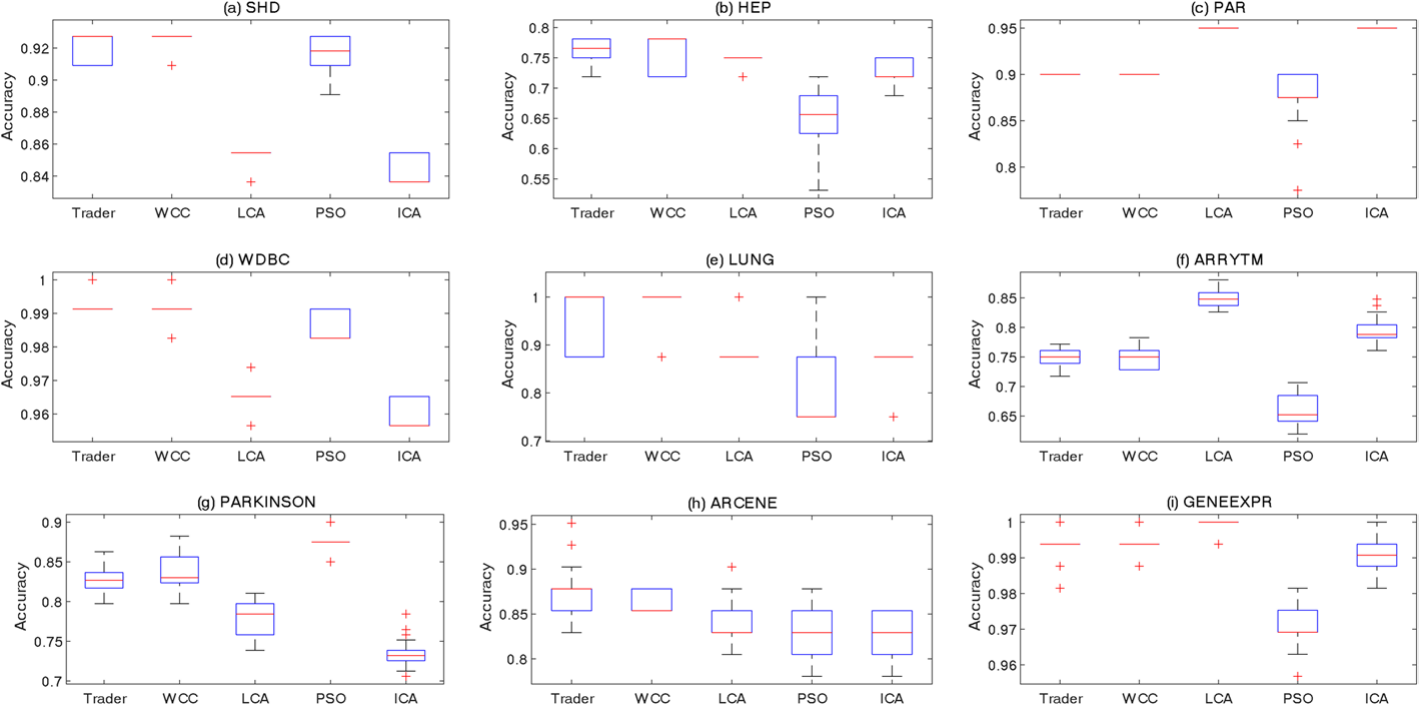
The stability behavior of the OAs on the **a** SHD, **b** HEP, **c** PAR, **d** WDBC, **e** LUNG, **f** ARRYTM, **g** PARKINSON, **h** ARCENE, and **i** GENEEXPR datasets on 50 individual executions

In every individual run, due to generating and changing CSs based on stochastic operators, the OAs selected a diverse subset of features and produced different values of accuracy. However, the final accuracy values must be similar to each other (the stability behavior of OAs) [37–40]. To examine this property of the OAs in detail, they were executed 50 times, and the distribution of their outputs was illustrated using boxplots (Fig. 4). The obtained outcomes indicated that *Trader* generally boosted the performance of SVM (via selecting the most informative features) and showed a more stable behavior than the other OAs.

Further, to investigate whether the performance of *Trader* and the other OAs was the same or not, the Wilcoxon rank-sum test was utilized. For this purpose, the results of *Trader* were considered as a test base and compared with the others’ outcomes (Table 2). It was observed that, for most cases, the p-value was less than 0.01 and about close to zero, resulting in rejecting the null hypothesis and validating that *Trader* outperformed the others in terms of enhancing the prediction power of SVM.

**Table 2:** The Wilcoxon rank-sum test of the OAs with *Trader’*s results as the test base

Dataset	WCC	LCA	PSO	ICA
LIV	1	1.68e−14	9.42e−04	1.68e−14
PID	1	2.70e−14	0.001	4.15e−14
SHD	1.52e−04	8.38e−13	0.103	4.52e−12
CHD2	2.35e−08	8.64e−14	1.98 e−04	1.19e−12
CHD5	4.35e−05	1.29e−12	2.41 e−04	4.28e−12
HEP	0.031	0.029	8.37e−11	8.65e−12
PAR	1.68e−14	1.68e−14	6.92e−07	1.68e−14
WDBC	2.76e−13	2.002e−13	9.16e−07	1.17e−12
LUNG	1.92e−04	1.81e−07	1.25e−09	5.69e−09
ARRYTM	0.061	2.14e−11	2.08e−11	5.37e−11
PARKINSON	0.078	1.18e−10	2.93e−11	2.59e−11
ARCENE	3.94e−04	1.81e−05	1.11e−07	1.29e−11
GENEEXPR	1	1	0.24	0.24

In the second part of the computational experiments, the proposed algorithm was embedded into a voting-based prediction system. To evaluate this system, different criteria were considered, such as accuracy (ACC), precision (PRE), sensitivity (SEN), specificity (SPC), and F-score (F). The outputs were then organized into several tables as well as receiver operating characteristic (ROC) and precision-recall (PR) curves. The compared machine learning approaches indicated different efficiencies on diverse datasets. Based on the outcomes, the proposed voting-based system generally outperformed the others in terms of the mentioned parameters (Table 3).

**Table 3:** Comparing the utilized methods based on the fivefold cross-validation technique

Dataset (size)	Algorithm	# selected features	Accuracy	Specificity	Precision	Sensitivity	F-SCORE
LIV 345 × 6	TRADER	4	**67.14**	**64.80**	**69.19**	**67.14**	**68.15**
	WCC	4	**67.14**	**64.80**	**69.19**	**67.14**	**68.15**
	LCA	2	60	55.15	77.23	60	67.53
	PSO	4	**67.14**	**64.80**	**69.19**	**67.14**	**68.15**
	ICA	4	62.86	58.68	73.10	62.86	67.59
	VOTING	4	**67.14**	**64.80**	**69.19**	**67.14**	**68.15**
PID 768 × 8	TRADER	4	76.77	**67.08**	76.15	76.77	76.46
	WCC	4	77.42	64.70	77.07	77.42	77.25
	LCA	4	77.4	67.42	76.84	73.55	77.13
	PSO	4	76.77	67.08	76.15	76.77	76.46
	ICA	3	74.19	63.02	73.32	74.19	73.75
	VOTING	4	**78.06**	65.94	**77.74**	**78.06**	**77.56**
CHD2 303 × 13	TRADER	5	**86.89**	**85.31**	**86.92**	**86.89**	**86.90**
	WCC	4	85.24	80.77	85.22	85.24	84.90
	LCA	5	86.88	80.04	87.87	86.88	87.39
	PSO	5	82.32	83.22	83.49	82.32	83.30
	ICA	5	85.24	85.92	85.95	85.24	85.34
	VOTING	5	**86.89**	**85.31**	**86.92**	**86.89**	**86.90**
CHD5 303 × 13	TRADER	5	63.93	61.43	61.62	63.93	62.76
	WCC	5	62.29	67.75	58.69	62.29	64.70
	LCA	5	63.93	66.23	62.83	63.93	65.12
	PSO	4	60.66	52.09	58.11	60.66	59.35
	ICA	4	62.29	64.59	58.05	62.29	63.32
	VOTING	5	**67.21**	**63.30**	**63.95**	**67.21**	**65.54**
SHD 270 × 13	TRADER	5	**92.73**	**89.43**	**92.82**	**92.73**	**92.77**
	WCC	5	90.90	88.38	90.84	90.90	90.89
	LCA	5	**92.73**	**89.43**	**92.82**	**92.73**	**92.77**
	PSO	5	89.09	87.34	89.09	89.09	89.09
	ICA	5	87.27	82.01	87.35	87.27	87.31
	VOTING	5	**92.73**	**89.43**	**92.82**	**92.73**	**92.77**
HEP 150 × 19	TRADER	6	**78.13**	**75.21**	**84.51**	**78.13**	**81.19**
	WCC	5	75	71.67	83	75	78.80
	LCA	6	68.75	66.15	71.11	68.75	69.91
	PSO	6	65.63	62.61	68.36	65.63	66.96
	ICA	6	71.88	70.48	72.34	71.88	72.11
	VOTING	6	**78.13**	**75.21**	**84.51**	**78.13**	**81.19**
PAR 197 × 22	TRADER	6	**90**	**70**	**91.18**	**90**	**90.58**
	WCC	6	**90**	**70**	**91.18**	**90**	**90.58**
	LCA	6	**90**	**70**	**91.18**	**90**	**90.58**
	PSO	6	80	40	84.21	80	82.5
	ICA	5	87.50	69.17	87.34	87.50	87.42
	VOTING	6	**90**	**70**	**91.18**	**90**	**90.58**
WBDC 569 × 31	TRADER	6	100	100	100	100	100
	WCC	6	100	100	100	100	100
	LCA	5	98.26	96.86	98.31	98.26	98.28
	PSO	6	95.65	95.11	95.65	95.65	95.65
	ICA	6	94.78	93.89	94.80	94.78	94.83
	VOTING	6	**100**	**100**	**100**	**100**	**100**
LUNG 32 × 56	TRADER	8	87.50	87.50	90	87.50	88.75
	WCC	8	87.50	98.21	93.75	87.50	87.50
	LCA	8	87.50	98.21	93.75	87.50	87.50
	PSO	10	62.5	72.50	73.75	62.5	62.5
	ICA	8	75.00	96.42	91.66	75.00	75.00
	VOTING	8	**100**	**100**	**100**	**100**	**100**
ARRYTM 452 × 279	TRADER	9	73.91	70.84	78.95	73.91	76.35
	WCC	7	77.17	72.75	76.90	77.17	77.17
	LCA	9	**82.60**	**77.61**	**82.84**	**79.34**	**82.09**
	PSO	9	64.13	63.68	64.09	64.13	64.11
	ICA	9	78.26	73.41	77.99	78.26	78.26
	VOTING	9	77.17	74.84	80.01	77.17	88.57
PARKINSON 756 × 754	TRADER	10	83.66	**52.95**	82.92	83.66	83.29
	WCC	9	82.35	46.53	81.78	82.35	82.7
	LCA	10	79.08	43.55	76.40	79.08	77.72
	PSO	10	77.12	22.88	70.92	77.12	73.89
	ICA	10	83.66	52.95	82.92	83.66	83.29
	VOTING	10	**84.31**	47.11	**86.96**	**84.31**	**85.62**
ARCENE 200 × 10,000	TRADER	10	**90.24**	**89.42**	**90.57**	**90.24**	**90.41**
	WCC	9	82.93	80.95	84.77	82.93	83.84
	LCA	8	**90.24**	**89.42**	**90.57**	**90.24**	**90.41**
	PSO	10	80.49	82.43	83.67	80.49	82.05
	ICA	10	87.80	86.82	87.82	87.80	87.81
	VOTING	10	**90.24**	**89.94**	90.24	**90.24**	90.23
GENEEXPR 801 × 20,531	TRADER	15	**100**	**100**	**100**	**100**	**100**
	WCC	15	**100**	**100**	**100**	**100**	**100**
	LCA	15	**100**	**100**	**100**	**100**	**100**
	PSO	14	95.23	**100**	**100**	90.90	95.23
	ICA	14	95.23	**100**	**100**	90.90	95.23
	VOTING	15	**100**	**100**	**100**	**100**	**100**

**Fig. 5:**
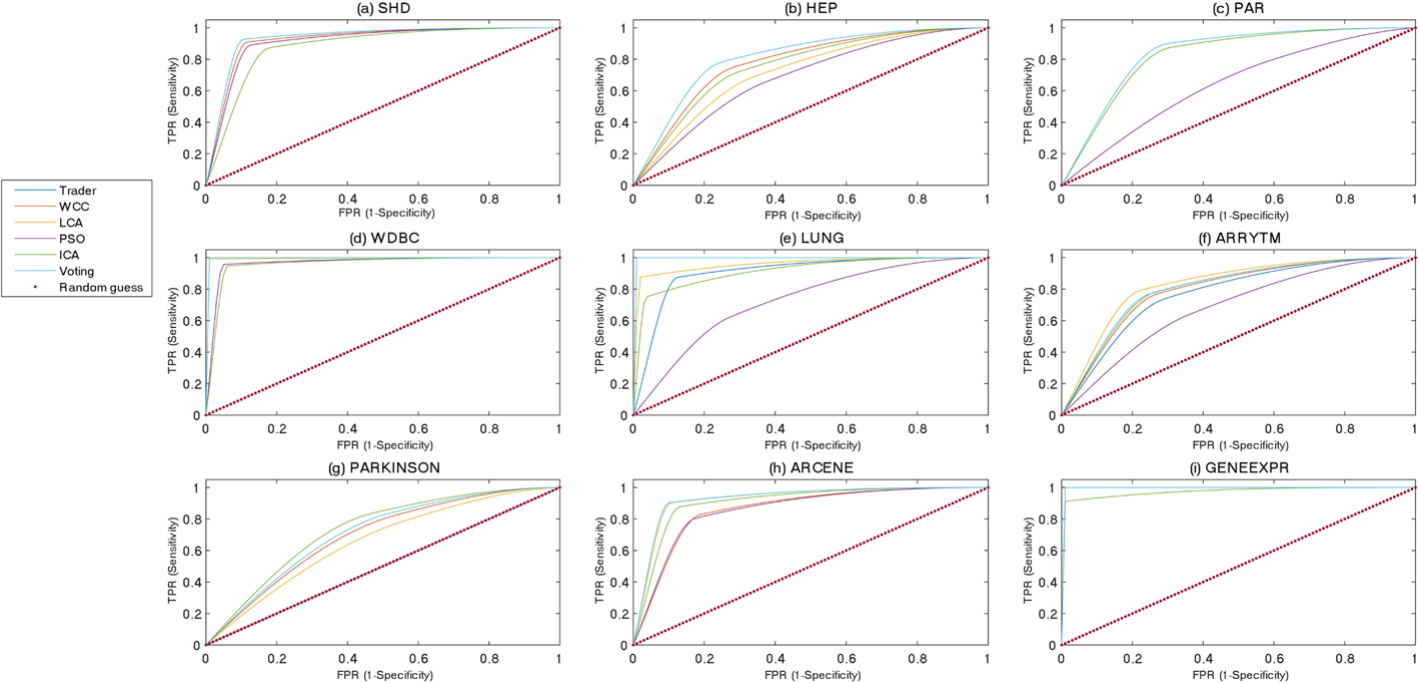
The ROC curve of the algorithms on the **a** SHD, **b** HEP, **c** PAR, **d** WDBC, **e** LUNG, **f** ARRYTM, **g** PARKINSON, **h** ARCENE, and **i** GENEEXPR datasets

**Fig. 6:**
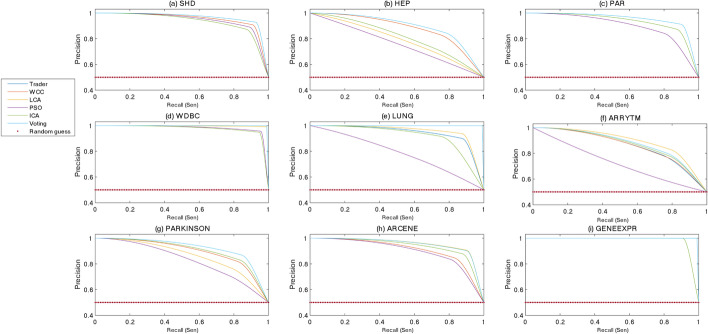
The PR curve of the algorithms on the **a** SHD, **b** HEP, **c** PAR, **d** WDBC, **e** LUNG, **f** ARRYTM, **g** PARKINSON, **h** ARCENE, and **i** GENEEXPR datasets

**Fig. 7:**
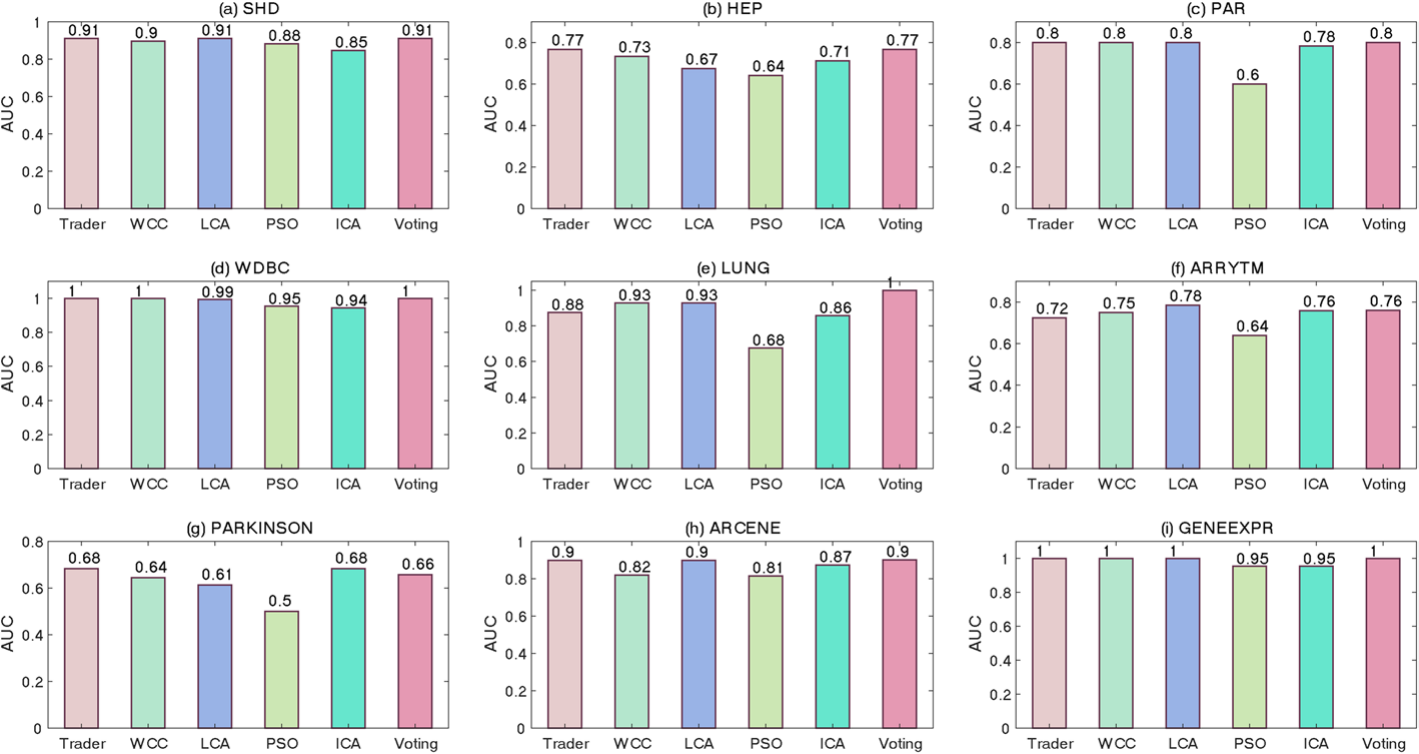
The AUC bar diagram for ROC curves on the **a** SHD, **b** HEP, **c** PAR, **d** WDBC, **e** LUNG, **f** ARRYTM, **g** PARKINSON, **h** ARCENE, and **i** GENEEXPR datasets

**Fig. 8:**
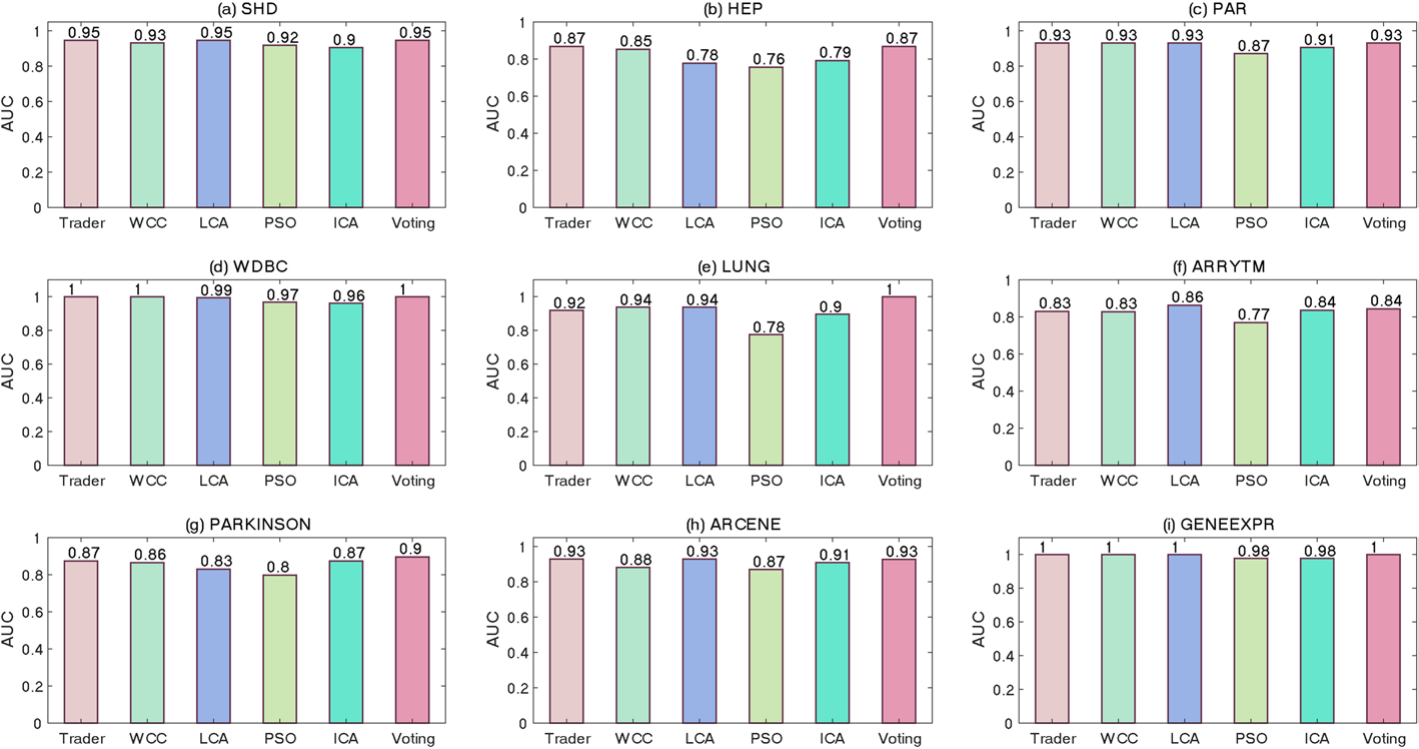
The AUC bar diagram for PR curves on the **a** SHD, **b** HEP, **c** PAR, **d** WDBC, **e** LUNG, **f** ARRYTM, **g** PARKINSON, **h** ARCENE, and **i** GENEEXPR datasets

The classification power of the generated models, separating positive or negative data samples into their related classes, was examined using the ROC and PR curves shown in Figs. 5 and 6, respectively. Although most of previously performed studies utilized the ROC and PR diagrams for evaluating the performance of binary classifiers, the presented study extended this concept to multi-class classifiers. To this end, Eq. 3 was used for calculating the values of SEN, SPC, and PRE. The acquired diagrams expressed that the proposed voting-based approach was closer to a perfect classifier than the others were. To display the area under curve (AUC) of the classifiers, for every dataset, two bar diagrams were provided, showing the AUC of the ROC (Fig. 7) and PR (Fig. 8) curves, respectively.

In the last part of the computational experiments, a comprehensive comparison between the proposed ML method and those suggested in the previous studies was performed. Up to the time of designing the present study, much attention has already been paid to introducing novel ML techniques and generating powerful biological data classifiers. Among these studies, the artificial neural network-based ML approach, introduced by Arabi et al., outperformed the other previously introduced ML methods in terms of the classification benchmarks. Here, the outputs of these studies were collected and then extended with the results obtained from the current study (Table 4). The outcomes indicated that the accuracy of the proposed voting-based ML approach was higher than the accuracy of the previously introduced ML methods. All in all, the suggested voting-based system improved all the criteria by ~ 10%.

**Table 4:** A comprehensive comparison between the proposed voting-based method and the other previously introduced approaches

Method name	Description	Accuracy				References
		WDBC	CHD5	CHD2	SHD	
Cooperative coevolution and RF	Filtering samples and features using the genetic algorithm and offering a clinical decision support system using random forest	97.1	–	93.4	96.8	[41]
ECSA	Extending crow search algorithm for feature selection and categorizing biological samples using the KNN algorithm	95.76	–	–	82.96	[42]
DISON and ERT	Providing a clinical decision support system using an extremely randomized tree-based feature selection algorithm and creating a prediction model using Diverse Intensified Strawberry Optimized Neural network	–	–	93.67	94.5	[43]
Adaboost SVM	Choosing informative features using three bioinspired optimization algorithm and Adaboost SVM	98.73	–	–	–	[44]
AGFS	Merging the genetic algorithm and fuzzy logic concept for classifying clinical datasets	–	**76.67**	–	–	[45]
SRLPSO-ELM	Proposing a self-adaptive machine learning technique based on the particle swarm optimization algorithm and extreme learning classifier	–	–	91.33	89.96	[46]
SVM-GA	Generating a clinical data classification model based on combining the genetic algorithm and the SVM classifier	–	72.55	90.57	–	[47]
ABCO with SVM	Employing the ant colony optimization algorithm for picking out features and evaluating them using the SVM classifier	–	–	83.17	84.81	[48]
CFCSA	Designing a hybrid system combining crow search optimization algorithm, chaos theory, and fuzzy c-means algorithm	98.6	–	–	88.0	[49]
CSA	Applying the crow search optimization algorithm for selecting features and creating a prediction model using the KNN algorithm	90.28	–	–	78.84	[50]
RS-BPNN	Building a prediction model for classifying clinical datasets using the rough set theory and backpropagation neural network	98.60	–	–	90.40	[51]
FELM	Extending the concept of fuzzy logic and extreme learning for training an artificial neural network	–	73.77	93.55	94.44	[52]
ANNWCC	Training an artificial neural network using the world competitive contests algorithm	–	71.5	**94.5**	**96.5**	[25]
CSO, KH, BFO, and super learner	Combining three optimization algorithms with the SVM classifier	96.83	–	84.00	86.36	[48]
TRADER -SVM	Selecting features using the *Trader* algorithm and evaluating them using the SVM classifier	**100**	64.96	88.85	89.45	–
Proposed voting-based model	Labeling a given data sample based on aggregating the prediction results of several models	**100**	67.21	88.85	92.73	–

## Discussion

To classify various types of clinical/biological datasets, the present study introduced a novel wrapper ML method that combined the *Trader* algorithm for selecting a near-optimal subset of features/genes and the SVM classifier for scoring them. Although the previous studies had suggested several ML methods and algorithms to stratify clinical/biological datasets [53–55], they encountered two critical limitations described as follows.

First, some literary works ignored the FS concepts in the data preprocessing step or utilized some heuristic filter-based FS techniques. For instance, several studies ranked and reduced the total number of the existing features/genes in a specific application such as introducing a limited number of genes as potential biomarkers for certain cancer [56]. To this end, some statistical-based FS algorithms have been utilized, such as the data Entropy-based FS method. As demonstrated in many recent bioinformatics-related pieces of research, wrapper-based FS approaches outperformed the filter and embedded-based FS techniques [57, 58], and two-step FS methods usually showed better functionality than single-step procedures [59–61]. Besides, in some cases, previous studies presented that filter-based FS techniques may reduce the prediction power of a learner [8, 62]. Hence, given the capabilities of the *Trader* algorithm in Np-hard problems, this study developed the algorithm for selecting the features and applied it not only to large-size datasets but also to small-size ones. The related outcomes (obtained from both the small and large-size datasets) indicated that the FS concept was a critical preprocessing step for biological applications, and the performance of the algorithms differed from each other on various datasets. In addition to gaining a suitable prediction model, the outputs of the FS phase may be essential for designing diagnosis/treatment plans, such as introducing the selected features/genes/proteins/miRNA as potential biomarkers for a wide range of diseases. The discovered biomarkers might be further investigated to determine their druggability properties and find candidate medicines to inhibit them.

Second, some previously carried out studies tested the usefulness of their methods on small-size datasets [63, 64]. Therefore, their proposed approaches could not be embedded into software packages due to their lower performances on large-size data. To address the mentioned restriction, a voting-based ML framework was introduced and applied to the different datasets having various properties. It was shown that the suggested framework could boost the prediction power of classification systems on both the small and large-size datasets whereas the previously introduced ML techniques lost their performances on large-size datasets. For example, Arabi et al*.* introduced and developed a perceptron-based artificial neural network for classifying 13 clinical/biological datasets and showed that their designed artificial neural network had a higher prediction ability than the other performed methods [25]. Arabi’s proposed method generated a distinct model for every class of a given data and categorized a data sample into a group whose related model represented the highest value of score. However, the outcomes of the present study exhibited that the mentioned ML method (introduce by Arabi et al.) suffered from overfitting on the small-size datasets. In other words, the proposed voting-based ML system yielded a more powerful prediction model on large-size datasets than the previously performed approaches. On the small-size datasets, the efficiency of the proposed ML framework was slightly lower than that of Arabi’s method in terms of the classification criteria. This issue was probably because of overfitting Arabi’s approach on the small-size datasets.

Like all the other previously carried out studies, the present work also might suffer from some limitations. Especially, the current study was organized based on the five OAs producing non-deterministic but acceptable outcomes. Hence, in designing a healthcare system, the deterministic rate may decrease. To deal with such a limitation, a possible solution can be identifying a proper configuration of algorithms that can correctly display a synergic effect. However, obtaining such a configuration seems to be a challenging task. Collectively, a combination of algorithms, such as various types of operators for changing CSs, might be an advantageous approach.

## Conclusion

This study extended our previously introduced optimization algorithm, *Trader*, to select a near-optimal subset of features/genes and proposed a voting-based machine learning technique to classify large-size biological/clinical datasets. According to the acquired results, it was indicated that the suggested voting-based classification framework yielded better predictions than the other previously performed studies. As a result, this technique can be considered an effective diagnosis/treatment approach such as discovering potential biomarkers and drugs to combat different diseases. In addition, the outcomes indicated that the feature selection concept is an essential preprocessing phase not only for large-size biological/clinical datasets but also for small-size ones, whereas most of the prior studies neglected the effect of the feature selection concept in their computational methods.

## Data Availability

The data of interest were obtained from the UCI machine learning repository (https://archive.ics.uci.edu/ml/index.php).

## Abbreviations

ACC: Accuracy
CS: Candidate solution
CHD: Cleveland heart disease
FS: Feature selection
IE: Importing-exporting
KNN: K-nearest neighbor
ML: Machine learning
OA: Optimization algorithm
PR: Precision-recall
PRE: Precision
ROC: Receiver operating characteristic
SEN: Sensitivity
SHD: Statlog heart disease
SPC: Specificity
SVM: Support vector machine
UCI: University of California, Irvine
WCC: World competitive contests
WDBC: Wisconsin diagnostic breast cancer
